# Functional evolution of scorpion venom peptides with an inhibitor cystine knot fold

**DOI:** 10.1042/BSR20130052

**Published:** 2013-06-27

**Authors:** Bin Gao, Peta J. Harvey, David J. Craik, Michel Ronjat, Michel De Waard, Shunyi Zhu

**Affiliations:** *Group of Animal Innate Immunity, State Key Laboratory of Integrated Management of Pest Insects and Rodents, Institute of Zoology, Chinese Academy of Sciences, 1 Beichen West Road, Chaoyang District, Beijing 100101, People's Republic of China; †Institute for Molecular Bioscience, University of Queensland, Brisbane, Queensland 4072, Australia; ‡Grenoble Institut des Neurosciences, Inserm U836, équipe 3, LabEx Ion Channel Science and Therapeutics, Bât. Edmond J. Safra, Université Joseph Fourier, Site Santé de la Tronche, BP 170, 38042 Grenoble Cedex 9, France

**Keywords:** cysteine stabilized α-helical and β-sheet motif, functional dyad, ryanodine receptor, scorpion toxin, solution structure, voltage-gated ion channel, CSαα, cysteine-stabilized helix-loop-helix, CSαβ, cysteine-stabilized α-helical and β-sheet, ECOSY, exclusive correlation spectroscopy, ICK, inhibitor cystine knot, λ-MK1, λ-MeuKTx-1, MCa, *Scorpio maurus palmatus*, RACE, 3′ rapid amplification of cDNA ends, SR, sarcoplasmic reticulum, UTR, untranslated region

## Abstract

The ICK (inhibitor cystine knot) defines a large superfamily of polypeptides with high structural stability and functional diversity. Here, we describe a new scorpion venom-derived K^+^ channel toxin (named λ-MeuKTx-1) with an ICK fold through gene cloning, chemical synthesis, nuclear magnetic resonance spectroscopy, Ca^2+^ release measurements and electrophysiological recordings. λ-MeuKTx-1 was found to adopt an ICK fold that contains a three-strand anti-parallel β-sheet and a 3_10_-helix. Functionally, this peptide selectively inhibits the *Drosophila Shaker* K^+^ channel but is not capable of activating skeletal-type Ca^2+^ release channels/ryanodine receptors, which is remarkably different from the previously known scorpion venom ICK peptides. The removal of two C-terminal residues of λ-MeuKTx-1 led to the loss of the inhibitory activity on the channel, whereas the C-terminal amidation resulted in the emergence of activity on four mammalian K^+^ channels accompanied by the loss of activity on the *Shaker* channel. A combination of structural and pharmacological data allows the recognition of three putative functional sites involved in channel blockade of λ-MeuKTx-1. The presence of a functional dyad in λ-MeuKTx-1 supports functional convergence among scorpion venom peptides with different folds. Furthermore, similarities in precursor organization, exon–intron structure, 3D-fold and function suggest that scorpion venom ICK-type K^+^ channel inhibitors and Ca^2+^ release channel activators share a common ancestor and their divergence occurs after speciation between buthidae and non-buthids. The structural and functional characterizations of the first scorpion venom ICK toxin with K^+^ channel-blocking activity sheds light on functionally divergent and convergent evolution of this conserved scaffold of ancient origin.

## INTRODUCTION

Potassium channels are a large and diverse family of ion channels that selectively transport K^+^ ions across membranes of excitable cells (neuronal and muscle tissues) and non-excitable cells [1]. They control the flow of K^+^ ions into and out of cells and thereby play key roles in generating action potentials and maintaining the resting membrane potential. Potassium channels regulate many key physiological processes, including neurotransmitter release, immune response, heart rate, insulin secretion, neuronal excitability, epithelial electrolyte transport, smooth muscle contraction and cell proliferation [2–4]. Voltage-gated potassium (K_v_) channels assemble as symmetric tetramers with each monomer containing six transmembrane α-helices (S1–S6) connected by five linker regions, in which S1–S4 constitute the voltage-sensor module at the periphery of the structure and S5–S6 form the ion-conducting pore in the centre [5]. Since K_v_ channel inhibition can greatly increase the excitability of neurons and results in rapid death of prey, this class of molecules becomes the major pharmacological targets of naturally occurring toxins from phylogenetically diverse venomous animals, including scorpions, spiders, snakes, cone snails and sea anemone [6–10].

Scorpions were among the first animals living on land about 420 million years ago. As the oldest venomous arachnids on earth, their venom has evolved a large number of gene-encoded peptide toxins that impair functions of Na^+^, K^+^ and Cl^−^ ion channels [11]. Interestingly, scorpions have used only 1-fold to develop their arsenal targeting Na^+^ and Cl^−^ channels, but repeatedly evolved multiple folds to capture diverse prey via K_v_ channels [12]. Peptides with a CSαβ (cysteine-stabilized α-helical and β-sheet) structural fold are the most predominant components of scorpion venom-affecting K_v_ channels, and are divided into three distinct groups: α-, β- and γ-KTxs [13]. The α-KTx group contains at least 26 subfamilies, its members being short-chain peptides of 23–42 amino acids with three or four disulfide bridges and specific inhibitory activity on K_v_ channels, as well as small-conductance Ca^2+^-activated K^+^ (SK_Ca_) channels and big-conductance Ca^2+^-activated K^+^ (BK_Ca_) channels. Most peptides in this group have a ‘functional dyad’ that directly interacts with the pore of *Shaker*-related K_v_ channels [14]. The β-KTx group consists of long-chain toxins of 50–75 amino acids with an extended N-terminus relative to α-KTxs [15]. Although pharmacological targets of most members remain to be characterized, it was found that TstβKTx is a high-affinity blocker of K_v_1.1 at nanomolar concentrations. In contrast, peptides belonging to the γ-KTx group have a similar size and a conserved 3D structure to α-KTxs, but they selectively interact with ERG channels on the turret region rather than the pore [16,17].

Apart from the KTxs mentioned above, scorpion venom also contains several minor components with different folds that affect K_v_ channels:

(1) κ-KTxs that have a scaffold of two parallel helices linked by two disulfide bridges [18].
(2) Peptides that possess a pattern of six cysteines similar to α-, β-, and γ-KTxs but adopt an unexpected CSαα (cysteine-stabilized helix-loop-helix)-fold [19]. Both the CSαα peptides and κ-KTxs are relatively poor blockers of K_v_ channels, with blocking activity only at high micromolar concentrations (40–200 μM) despite the presence of a putative functional dyad.
(3) Peptides that share a common cysteine pattern with animal venom-derived Kunitz-type K^+^ channel toxins (e.g. Hg-1, BmKTT-1 and BmKTT-2) [20]. Most of these peptides have bi-functional properties and inhibit both K_v_ channels and proteases.
(4) Peptides sharing a common cysteine pattern with arthropod venom-derived ICK (inhibitor cystine knot)-type toxins. ImKTx1 is the first member identified as a blocker of K_v_1.1–K_v_1.3 [21]; however, its 3D structure and genomic organization remain unsolved, which leaves uncertainty in evolutionary relationship with other ICK peptides with different biological activities and diverse sequences.

The ICK fold is an exceptionally stable structural motif comprising an embedded ring formed by two disulfide bridges (Cys^1^–Cys^4^ and Cys^2^–Cys^5^) and their connecting backbone segments, which is threaded by a third disulfide bridge (Cys^3^–Cys^6^) [22]. This motif defines a large superfamily of polypeptides derived from plants, fungi, invertebrates and even viruses, whose members possess diverse biological activities [23]. To date, maurocalcine (*Scorpio maurus palmatus*, abbreviated as MCa) and imperatoxin A (*Pandinus imperator*) are the two most thoroughly studied scorpion venom ICK peptides and have been characterized as activators of sarcoplasmic reticulum Ca^2+^ release channels/ryanodine receptors of skeletal and cardiac muscles [24,25].

In this work, we describe a new scorpion ICK peptide, named λ-MeuKTx-1 (abbreviated as λ-MK1) that is designated as the first member of the λ-KTx family coming from *Mesobuthus eupeus* in terms of its genomic organization, solution structure, biological activity and structure–function relationship. Our work provides new evidence in favour of divergent evolution between scorpion venom ICK-type K^+^ channel inhibitors and Ca^2+^ release channel activators and functional convergence among λ-KTxs and other toxins with different folds.

## MATERIALS AND METHODS

### Isolation of cDNA and genomic clones

**Figure 1:**
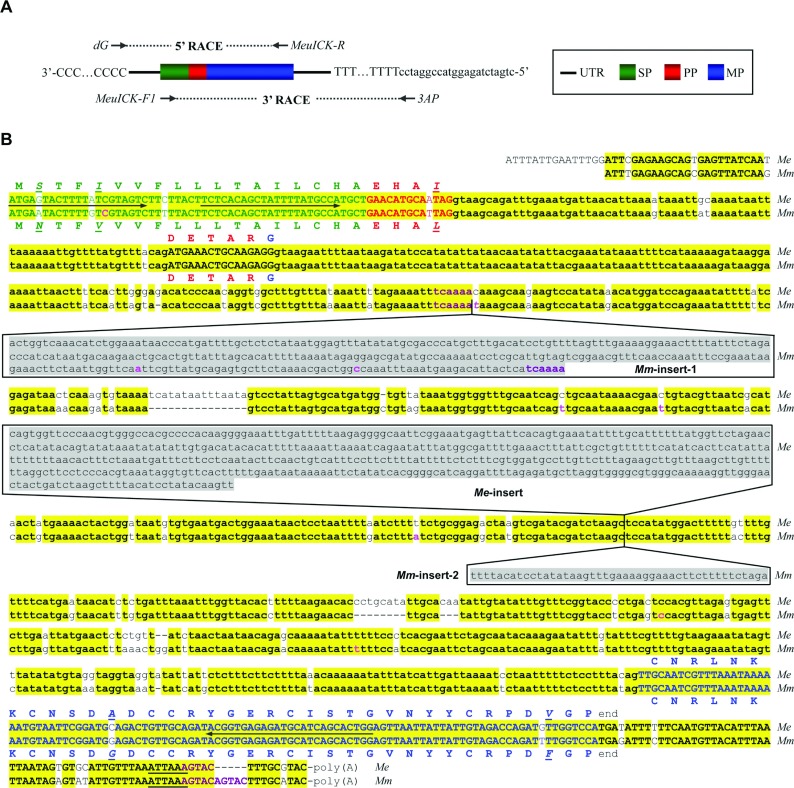
Molecular cloning of λ-MK1 (**A**) PCR strategy for isolating *λ-MK1* cDNA from the *M. eupeus* venom gland. UTR, untranslated region; SP, signal peptide; PP, propeptide; MP, mature peptide; (**B**) Comparison of nucleotide and deduced amino acid sequences of *λ-MK1* and its orthologous gene *BmCa1*. *Mm*, *Mesobuthus martensii*; *Me*, *Mesobuthus eupeus*. Exons and introns are shown by upper and lower letters, respectively. SP-, PP- and MP-coding regions and the corresponding amino acid sequences are shown in different colours. Identical nucleotide and non-identical amino acid sequences are shadowed in yellow and italicized/underlined, respectively. Three insertions in the intron regions are shadowed in grey and short repeats are shown in brown. The poly(A) signal (ATTAAA) is underlined once. *BmCa-1* was cloned and sequenced in this work, whose sequence shows nine polymorphic sites different from the previously reported sequence (GenBank® accession number DQ206446), indicated in pink. Arrows indicate positions of primers and their sequences are provided in Supplementary Table S1 (available at http://www.bioscirep.org/bsr/033/bsr033e047add.htm).

Total RNA and genomic DNA of *M. eupeus* and *M. martensii* were prepared according to the previously described method [26]. Reverse-transcription of total RNA into the first-strand cDNA was performed by an RT-PreMix kit (SBS Genetech) and a universal dT3AP [oligo(dT)-containing adaptor primer], which were directly used as templates for RACE (3′ rapid amplification of cDNA ends) with primers MeuICK-F1 and 3AP (Figure 1A). Purified first-strand cDNAs were tailed and used as templates for 5′ RACE with primers dG and MeuICK-R (Figure 1A). To determine the exon–intron organization of *λ-MK1*, a specific primer (MeuICK-F2) was synthesized to amplify genomic DNA in combination with MeuICK-R. PCR products were ligated into the pGEM-T Easy vector and recombinant plasmids were transformed into *Escherichia coli* DH5α. Positive clones were sequenced with T7 or SP6 primer. Nucleotide sequences of cDNA and genomic DNA clones of *λ-MK1* and a polymorphic clone of *BmCa-1* with nine site mutations have been deposited in the GenBank® database (http://www.ncbi.nlm.nih.gov/) under accession numbers of GU187948, EF445101 and DQ272503. All the primers used here are listed in Supplementary Table S1 (available at http://www.bioscirep.org/bsr/033/bsr033e047add.htm).

### Chemical synthesis and oxidative refolding

λ-MK1 and λ-MK1_a_ in their reduced form were chemically synthesized by ChinaPeptides Co., Ltd. and Scilight-Peptide Inc., respectively. For oxidative refolding, peptide samples were dissolved in 0.1 M Tris–HCl buffer (pH 8.0) to a final concentration of 0.5 mM and incubated at 25°C for 48 h. Peptides were purified by RP-HPLC (reversed-phase HPLC). Purity and molecular masses of peptides were determined by MALDI-TOF-MS on a Kratos PC Axima CFR plus (Shimadzu Co. LTD).

### CD spectroscopy

The JASCO J-720 spectropolarimeter (Jasco) was used to record CD spectra of λ-MK1, λ-MK1-GP and λ-MK1_a_, which were measured in water at room temperature (25°C) from 260 to 190 nm by using a quartz cell of 1.0-mm thickness. Data were collected at 0.5 nm intervals with a scan rate of 50 nm/min.

### Heavy SR (sarcoplasmic reticulum) vesicles preparation

Heavy SR vesicles were prepared following a modified method of Kim and collaborators as described in Marty *et al.* [27]. Protein concentration was measured by the Biuret method.

### [^3^H]-ryanodine-binding assay

Heavy SR vesicles (1 mg/ml) were incubated at 37°C for 2 h 30 min in a buffer containing 5 nM [^3^H]-ryanodine, 150 mM NaCl, 2 mM EGTA, variable concentrations of CaCl_2_ to adjust pCa (-log[Ca^2+^]) at 7 or 5 and 20 mM Hepes, pH 7.4. λ-MK1 or MCa were added to the assay buffer just prior to the addition of heavy SR vesicles. [^3^H]-ryanodine bound to heavy SR vesicles was measured by filtration through Whatmann GF/B glass filters followed by three washes with 5 ml of ice-cold washing buffer composed of 150 mM NaCl, 20 mM Hepes, pH 7.4. Total radioactivity retained on the filter was measured by scintillation spectrometry. Non-specific binding was measured in the presence of 20 μM cold ryanodine. Specific [^3^H]-ryanodine was calculated by subtracting non-specific binding to the total binding. Each experiment was performed in triplicate and repeated at least two times. All data are presented in cpm (counts per minute) as mean±S.E.

### Ca^2+^ release measurements

Ca^2+^ release from heavy SR vesicles was measured using the Ca^2+^-sensitive dye, antipyrylazo III as described in Estève *et al.* [28] in a buffer containing: 100 mM KCl, 7.5 mM sodium pyrophosphate, 20 mM potassium Mops, pH 7.0, supplemented with 250 μM antipyrylazo III, 1 mM ATP/MgCl_2_, 5 mM phosphocreatine and 12 μg/ml creatine phosphokinase [29]. Ca^2+^ loading was started by three sequential additions of 20 μM CaCl_2_. In these loading conditions, no calcium-induced calcium release interferes with the observations.

### NMR spectroscopy and structure calculations

λ-MK1_a_ (1.0 mg) was dissolved in either 10% ^2^H_2_O/90% H_2_O or 100% ^2^H_2_O at pH ~3. Spectra were recorded on a Bruker Avance-600 spectrometer at 298 K or 283–298 K for variable temperature experiments. Solvent suppression was achieved using excitation sculpting with gradients [30]. 2D NMR experiments included TOCSY [31] using an MLEV-17 spin lock sequence [32] with 80 ms mixing time, NOESY (nuclear Overhauser enhancement spectroscopy) [33] with 200 ms mixing time, DQF-COSY (double-quantum-filtered correlation spectroscopy) [34], ECOSY (exclusive correlation spectroscopy) [35] and ^13^C HSQC (heteronuclear single-quantum coherence) experiments [36]. Spectra were acquired with 4096 complex data points in F2 and 512 increments in the F1 dimension, and were processed using TopSpin (Bruker Biospin) software. Chemical shifts were referenced to internal 2,2-dimethyl-2-silapentane-5-sulfonate. Processed spectra were analysed and assigned using the program SPARKY (http://www.cgl.ucsf.edu/home/sparky/).

The 3D structure was calculated by deriving distance and angle restraints from the NOESY, DQF-COSY and ECOSY spectra. In addition, backbone phi and psi dihedral angle restraints were generated from C_α_ and C_β_ chemical shifts using the program TALOS+ [37]. A family of structures consistent with the experimental restraints was calculated using the programs CYANA [38] and CNS [39]. Preliminary structures were calculated using a torsion angle simulated annealing protocol within CYANA and used to resolve ambiguities in the NOE assignments. Hydrogen bond restraints for slowly exchanging amides were added also. Disulfide bond restraints (with connectivity among resides 2–16, 9–22 and 15–31) were confirmed using the preliminary structures and the program PADLOC [40]. Once a complete set of input restraints (distance, hydrogen bond and dihedral angle restraints) was determined, the final structural family was generated within CNS using protocols from the RECOORD database [41] and the force field distributed with Haddock 2.0 [42]. A set of 50 structures was analysed for stereochemical quality using MOLPROBITY [43] and the 20 structures with lowest energy and best quality were chosen to represent the solution structure of λ-MK1_a_.

### Expression of K_v_ channels in *Xenopus* oocytes and two-electrode voltage-clamp recording

For the expression of K_v_ channels (rK_v_1.1–rK_v_1.4 and *Shaker* IR) and Na_v_ channels (rNa_v_1.1, rNa_v_1.2, rNa_v_1.4, rNa_v_1.5 and mNa_v_1.6) in *Xenopus* oocytes, the linearized plasmids were transcribed with the T7 or SP6 mMESSAGE-mMACHINE transcription kits (Supplementary Table S2, available at http://www.bioscirep.org/bsr/033/bsr033e047add.htm). Oocytes were digested for 1–2 h at room temperature in Ca^2+^-free ND96 (in mM: NaCl, 96; KCl, 2; CaCl_2_, 1.8; MgCl_2_, 2 and Hepes, 5, adjusted to pH 7.4 with NaOH) containing 0.5 mg/ml collagenase (type I, sigma). Oocytes were injected with 0.5 ng of cRNA by NANOLITER 2000 (World Precision Instruments Inc.). The oocytes were incubated in ND96 solution supplemented with 50 mg/l gentamycin sulfate at 18°C.

Ion channel currents were recorded 1–3 days after injection with Oocyte Clamp OC-725C (Warner Instument Corp.) and Digidata 1440A (Axon CNS, Molecular Devices) controlled by pCLAMP10 software (Axon). Pipettes were pulled by P-97 FLAMING/BROWN Micropipette Puller (Sutter Instrument Co.) with resistance of 0.1–1.0 MΩ when filled with 3 M KCl. rK_v_1.1–rK_v_1.4 and *Shaker* currents were evoked by 250 ms depolarization to 0 mV followed by a 250 ms pulse to −50 mV, from a holding potential of −90 mV. rNa_v_1.1, 1.2, 1.4, 1.5 and mNa_v_1.6 currents were evoked by 100 ms depolarization to −10 mV from a holding potential of −90 mV. All data were recorded from at least three independent oocytes (*n*≥3) and are presented as mean±S.E.. Leak subtraction was carried out with a P/4 protocol. Data were analysed by pCLAMP 10 (Molecular Devices) and SigmaPlot 10.0 (Systat Software).

### Structural modelling

The experimental structure of MCa (pdb entry 1C6W) was selected as the template to build the models of two bacterial ICK peptides (*Kr*ICK and *Ho*HP-ICKD) by SWISS-MODEL, a fully automated protein structure homology-modelling server (http://swissmodel.expasy.org/). Model quality was evaluated by Verify3D.

## RESULTS AND DISCUSSION

### Molecular characterization of λ-MK1

By using a combined technique of 3′ and 5′ RACE (Figure 1A), we isolated the full-length cDNA of *λ-MK1*, which includes a 5′ UTR (untranslated region) of 40 bp, an ORF of 195 bp and a 3′ UTR of 63 bp, with a polyadenylation site (ATTAAA) 13 nucleotides upstream of the cleavage site. The ORF codes for a precursor of 64 amino acids with three distinct domains: a signal peptide of 18 residues, a small anionic propeptide of nine amino acids terminated by a typical prohormone-processing signal (arginine) and a C-terminal part comprising the mature peptide of 37 residues (Figure 1B). *λ-MK1* is an othologous gene of *BmCa-1* in *Mesobuthus martensii* [44], whose structure and function are not solved yet. In comparison with BmCa-1, the precursor of λ-MK1 has five amino acid substitutions, of which three are located in the prosequence and two in the mature peptide.

**Figure 2:**
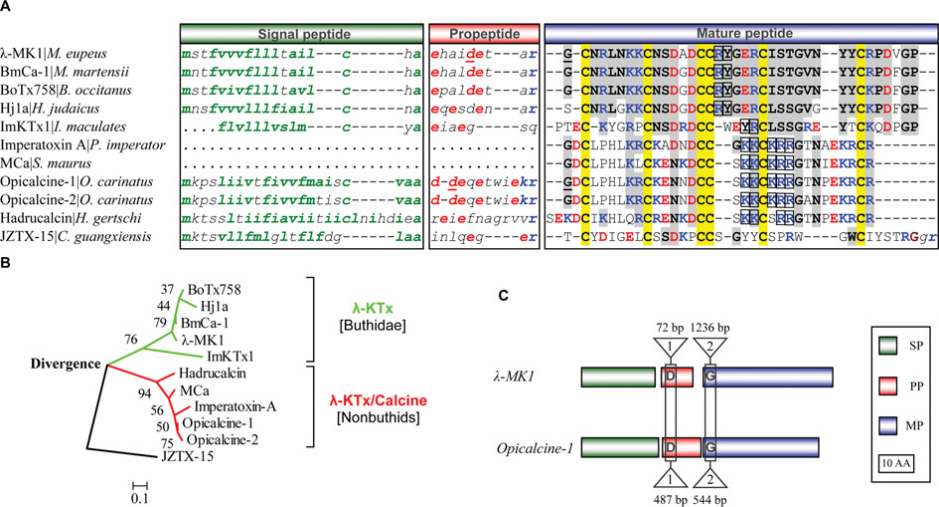
Scorpion venom ICK peptides (**A**) Amino acid sequences of precursors. Hydrophobic residues in SPs and acidic and basic residues in PPs and MPs are shown in green, red and blue, respectively. Amino acids disrupted by one intron are underlined once. Six conserved cysteines are shadowed in yellow and identical residues to λ-MK1 in grey. JZTX-15 is a spider ICK peptide from *Chilobrachys guangxiensis*. Sequence source: JZTX-15 (ABY71684); BmCa-1 (AF419253; BoTx758 (ACJ23131); Hj1a (ADY39527); Opicalcine-1 (AAP73822); Opicalcin-2 (P60253); Hadrucalcin (ACC99422); ImKTx1 [21]; MCa (P60254); Imperatoxin A (P59868); (**B**) An NJ tree rooted with JZTX-15, a spider ICK peptide (accession No. EU233865), which was reconstructed by using the mature amino acid sequences in Figure 2(**A**). Two distinct clades are shown in different colours. Numbers above branches are bootstrap values and the scale bar indicates total amino acid divergence; (**C**) Gene structure similarity between *λ-MK1* and *opicalcine-1* [23]. 1 and 2 represent intron phases.

*λ-MK1* is a split gene that contains three exons and two introns. The first intron (phase-1) of 72 bp interrupts the propeptide-coding region and the second intron (phase-2) of 1236 bp disrupts the first codon of the mature-peptide-coding region. Although *λ-MK1* and its orthologous gene *BmCa-1* share highly similar exon sequences, extensive divergence occurs in the second intron. Relative to *λ-MK1*, *BmCa-1* contains a unique insertion of 314 bp (herein called *Mm*-insert-1) in that its two ends have a six nucleotide direct repeat (TCAAAA), evidence for a mobile element [45]. A highly diversified region in size was also recognized between *λ-MK1* and *BmCa-1*: the former has 494 bp (termed *Me*-insert) whereas the latter only has 45 bp (*Mm*-insert-2) (Figure 1B). Database searches with λ-MK1 as the query identified BmCa-1 and two other uncharacterized scorpion toxin-like peptides (BoTx758 from *Buthus occitanus israelis* and Hj1a from *Buthotus judaicus*) as significant matches (Figure 2A). Several other hits with low sequence similarity were also retrieved. All these sequences derive from spiders and have an identical precursor organization and six conserved cysteines to λ-MK1 (results not shown). However, such searches failed to find previously known scorpion venom ICK peptides with Ca^2+^ release channel activating activity (e.g. MCa, opicalcines and imperatoxin A) [23–25] because of their highly dissimilar sequences.

### λ-MK1 is a new member of the scorpion ICK family

To date, a total of eight scorpion venom peptides with an ICK motif have been described or deposited in the GenBank® database, of which two were structurally identified (imperatoxin A and MCa) and four were functionally identified as either a K_v_ channel blocker (ImKTx1) [21] or Ca^2+^ release channel activators (imperatoxin A, MCa and hadrucalcin) [23,24,46]. Figure 2A shows precursor sequences of λ-MK1 and other ICK peptides from scorpions. With the exceptions of imperatoxin A and MCa whose gene sequences are unknown, other members possess a common precursor organization including signal peptide, propeptide and mature peptide, which is similar to those of U1-LITX-Lw1a and OcyC10, two insect-selective scorpion toxins with a DDH (disulfide-directed β-hairpin) fold [47], but differ from the most scorpion toxins with a CSαβ structural motif [48]. The amino acid consensus sequence of scorpion venom ICK peptides was determined to be CX_6_CX_4_DCCX_2–4_K/RCX_3_GX_4–6_CK/R (X, any amino acid) with a ring size from 13 to 15 amino acids.

According to sequence similarity and phylogenetic analysis, scorpion ICK peptides can be divided into two distinct subgroups. Peptides within a subgroup are highly conserved, showing up to 60% sequence identity, whereas peptides from different subgroups show greater divergence (only approximately 20% identity) (Figures 2A and 2B). Considering the functional data previously reported and described here that members coming from these two subgroups have K^+^ channel-blocking activity while only members from the second subgroup have Ca^2+^ release channel activating function, we name the former λ-KTx and the latter λ-KTx/calcine. Despite low sequence similarity, members coming from these two subgroups possess a completely identical exon–intron structure (Figure 2C) with a positionally conserved phase-1 intron disrupting the codon of an Asp at the propeptide region and a phase-2 intron splitting the codon of a Gly at the N-terminal region of the mature peptide, which provides strong evidence in favour of their common evolutionary origin.

### Characterization of synthetic λ-MK1

**Figure 3:**
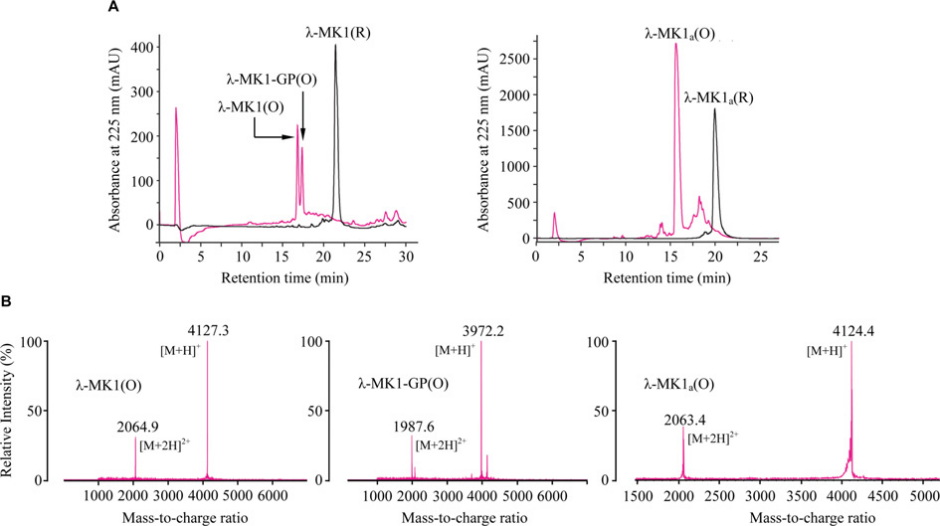
Oxidative refolding and identification of chemically synthetic λ-MK1 and λ-MK1_a_ (**A**) RP-HPLC showing retention time (*T*_R_) difference between the reduced (R) and oxidized (O) peptides. C_18_ column was equilibrated with 0.1% (w/v) TFA (trifluoroacetic acid) and purified proteins were eluted from the column with a linear gradient from 0 to 60% acetonitrile in 0.1% (w/v) TFA within 40 min; (**B**) MALDI-TOF MS of the oxidized peptides. The two main peaks in each spectrum correspond to the singly and doubly protonated forms of these peptides.

Under the chromatographic condition used here, the synthetic reduced λ-MK1 contains a single peak with more than 95% purity that was eluted at 22 min and its oxidation products consists of two major peaks eluting at 16.5 and 17.5 min, respectively (Figure 3A). Analysis of the two peaks by MALDI-TOF gave molecular masses of 4127.3 Da for the former and 3972.2 Da for the latter, which match 4125.64 Da, a calculated mass for the full-length λ-MK1 and 3971.47 Da for a mutant (named λ-MK1-GP) with two C-terminal residues (Gly^36^–Pro^37^) deleted (Figure 3B). This analysis indicates that six hydrogen atoms have been removed when three disulfide bridges are formed during oxidization. Earlier elution for the oxidized peptides is probably due to more hydrophobic residues becoming less accessible in the structured molecule when three disulfide bridges are formed. The deletion of the two residues from the synthetic peptide under oxidation and refolding suggests the instability of a peptide bond between Val^35^ and Gly^36^ in an alkaline environment. The instability of some peptide bonds (e.g. Asp–Pro, Asp–Gly, Asp–Ser) due to non-enzymatic hydrolysis in a specific pH range has been reported previously [49].

**Figure 4:**
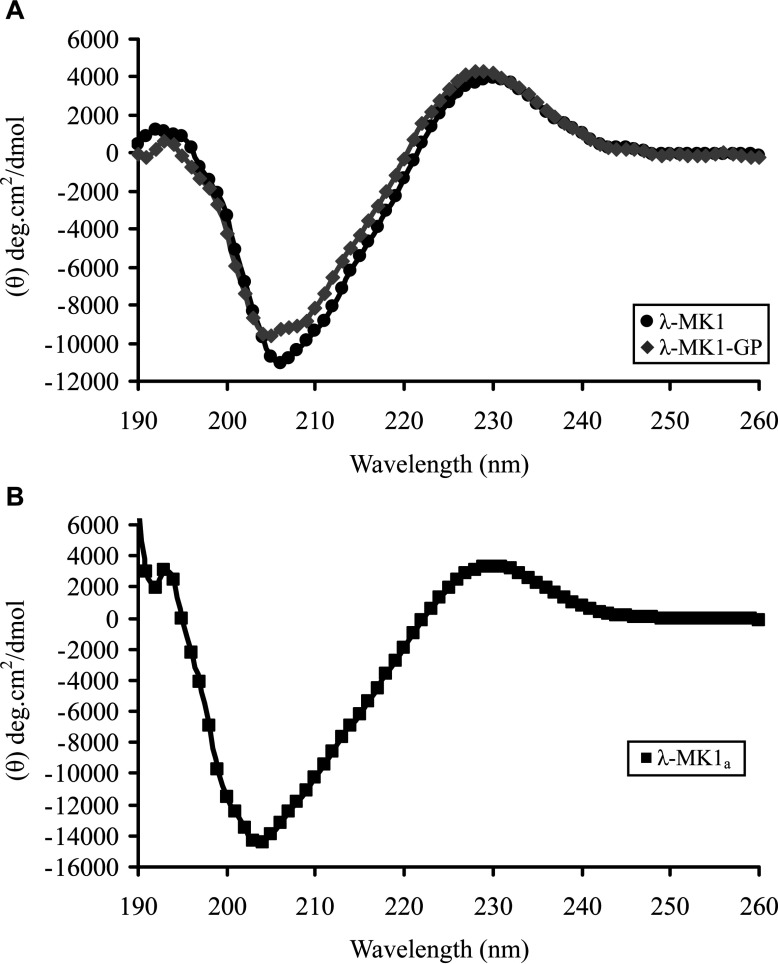
CD spectra of λ-MK1, λ-MK1-GP and λ-MK1_a_ Peptides are dissolved in H_2_O at a concentration of 0.3 mg/ml. Data are expressed as mean residue molar ellipticity (θ).

λ-MK1 and λ-MK1-GP both exhibit a similar CD profile, as characterized by a negative minimum around 205 nm and a strong positive band around the 228 nm region (Figure 4A). Such CD spectrum has also been observed in the tarantula toxin jingzhaotoxin-III [50], an Na^+^ channel blocker with an ICK fold, indicating that they possess a similar content of secondary structure elements and the deletion of the two residues did not alter the secondary structure of λ-MK1.

### Pharmacological targets of λ-MK1

**Figure 5:**
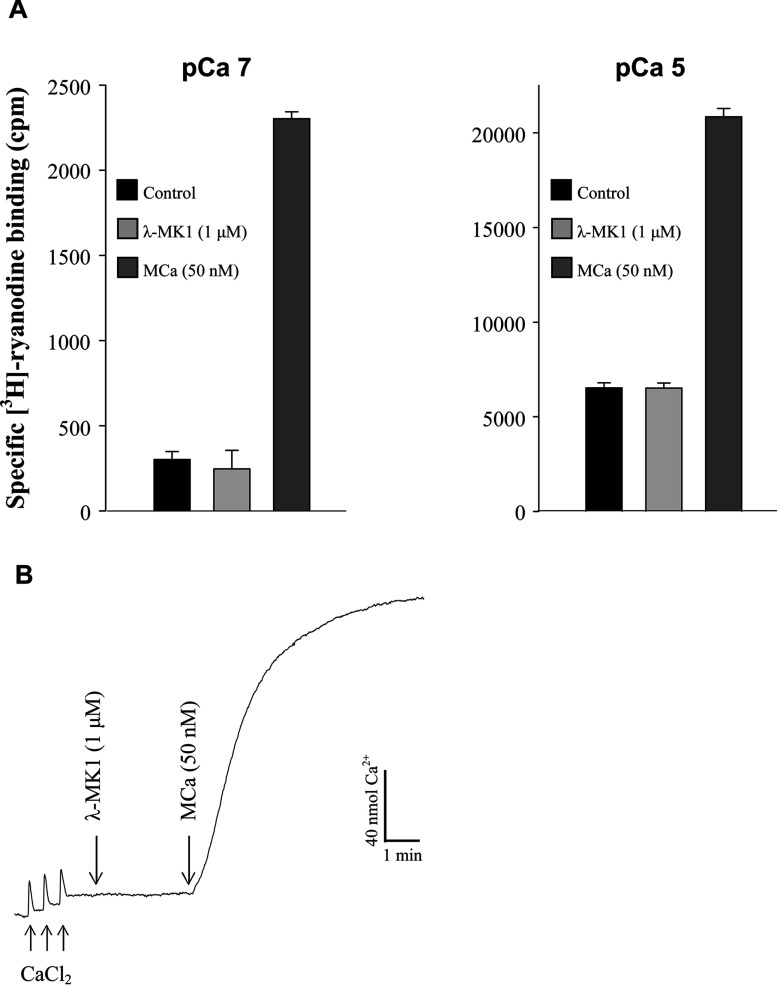
Evaluation of the activity of λ-MK1 on Ca^2+^ release channels/ryanodine receptors (**A**) [^3^H]-ryanodine binding assay. Specific [^3^H]-ryanodine binding on SR vesicles was measured at pCa 7 (left panel) and pCa 5 (right panel) in the presence of 5 nM [^3^H]-ryanodine. Control corresponds to the specific binding measured in the absence of toxin. In order to ascertain the functional state of the SR membrane preparation, we tested the effect of MCa on ryanodine binding in identical conditions; (**B**) Ca^2+^ release measurements. Heavy SR vesicles were actively loaded with Ca^2+^ by three additions of 20 μM (final concentration) of CaCl_2_ in the monitoring chamber. In these conditions, addition of λ-MK1 (1 μM final concentration) does not produce any Ca^2+^ release. In contrast, addition of 50 nM MCa induces strong Ca^2+^ release.

**Figure 6:**
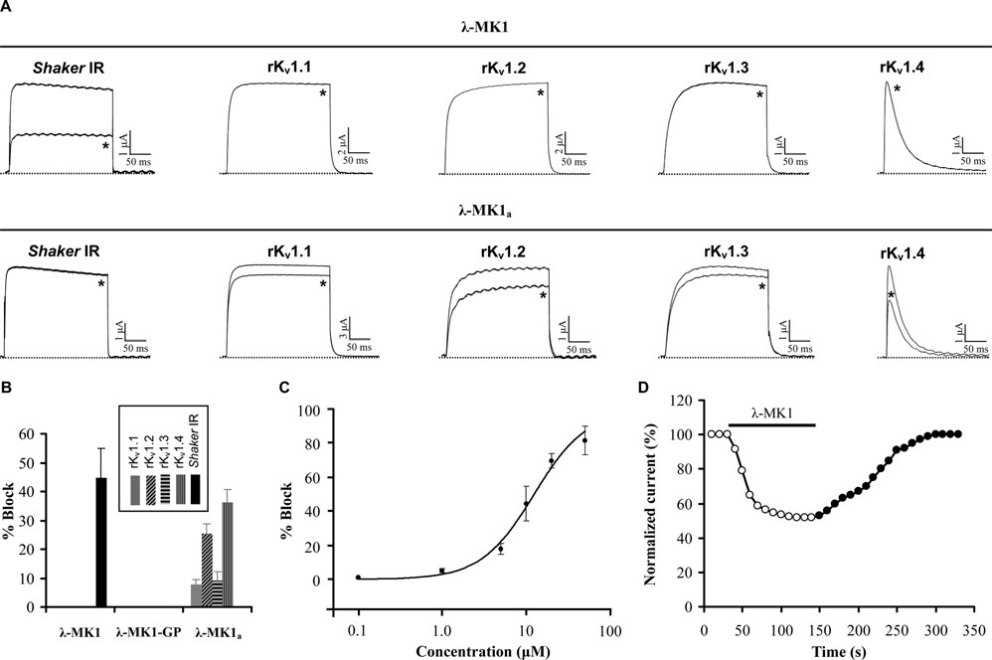
λ-MK1 and λ-MK1_a_ differentially inhibits K_v_ channels expressed in *Xenopus* oocytes (**A**) Representative whole-cell current traces in control and peptide conditions are shown. The dotted line indicates the zero-current level. The asterisk marks steady-state current traces in the presence of 10 μM peptides. Traces shown are representative traces of 3 independent experiments (*n*=3); (**B**) Blocking effects of λ-MK1 and its mutants with carboxyl terminal modifications on five cloned K_v_ channels expressed in *Xenopus* oocytes. The peptide concentration used here is 10 μM and data are presented as mean±S.E. (*n*=3); (**C**) The dose-response curve of λ-MK1 on the *Shaker* channel obtained by plotting the percentage blocked current as a function of increasing toxin concentrations. Each point represents mean±S.E. (*n*=3). These data points were fitted according to the Hill equation; (**D**) Kinetics of inhibition and reversibility of λ-MK1 on the *Shaker* channel. Fast inhibition and reversibility of the inhibition upon washout is shown by open circles and black circles, respectively.

Given a common origin between λ-MK1 and MCa, we firstly evaluated the effects of λ-MK1 on Ca^2+^ release channels/ryanodine receptors by [^3^H]-ryanodine-binding assays and Ca^2+^ release measurements. The results indicate that λ-MK1 is not an effector of the skeletal-type ryanodine receptor as it neither affects ryanodine binding on skeletal-type Ca^2+^ release channels/ryanodine receptors (Figure 5A) nor induces Ca^2+^ release from heavy SR vesicles (Figure 5B). To find possible pharmacological targets of λ-MK1, we screened a panel of ion channels expressed in *Xenopus* oocytes by electrophysiological measurements, which include five K_v_ channels (rK_v_1.1–rK_v_1.4, and the *Drosophila Shaker* channel) and five Na_v_ channels (rNa_v_1.1, rNa_v_1.2, rNa_v_1.4, rNa_v_1.5 and mNa_v_1.6). The results showed that the peptide selectively reduced the *Shaker* channel currents up to 44.7±10.2% at 10 μM (Figures 6A and 6B) with a peptide concentration at half-maximal efficacy (IC_50_) of 12.30±0.96 μM (Figure 6C), but had no effect on other K_v_ channels (Figures 6A and 6B) and the Na_v_ channels (results not shown). The affinity of λ-MK1 on the *Shaker* channel is comparable with that of several other scorpion toxins [18,19] and APEKTx1, a sea anemone peptide with Kunitz-type protease inhibiting property, on the mutated K_v_1.1 channels [51]. Blockade of the *Shaker* channel occurred rapidly and binding was reversible upon washout (Figure 6D). Overall, λ-MK1 did not alter the original shape of currents, suggesting that it represents a pore blocker of insect K_v_ channels. Phyletic selectivity of λ-MK1 is consistent with the observation that insects are the primary prey of *Mesobuthus eupeus*. Similarly, α-scorpion toxins from this species also essentially modulate insect rather than mammalian Na^+^ channels [52].

The deletion of Gly^36^ and Pro^37^ led to the loss of activity of λ-MK1 on the *Shaker* channel (results not shown), supporting a functional role of the carboxyl-terminus in interacting with the channel pore. This is further strengthened by the evolutionary conservation of these two residues in all the members of λ-KTx subgroup. To further investigate the functional significance of this region, we carried out C-terminal amidation, a common post-translational modification for many toxins from venoms of scorpions, spiders and cone snails [18,53,54]. The amidated peptide (named λ-MK1_a_) was oxidized and had a MALDI-TOF mass of 4124.4 Da, six Da smaller than the calculated mass of 4130.64 Da, suggesting that six hydrogen atoms were removed to form three disulfide bridges (Figure 3B). Intriguingly, such modification switched the target of λ-MK1 from the *Shaker* channel to mammalian K_v_ channels (Figures 6A and 6B). At 10 μM concentration, current reductions of rK_v_1.1 to rK_v_1.4 were 7.7±1.8, 25.3±3.6, 9.0±3.2 and 35.7±4.26% (*n*=3), respectively. This experiment further highlights the functional importance of the C-terminus of λ-MK1. In a prior study it was also shown that the same modification was able to increase the affinity of leiurotoxin I, a scorpion α-KTx, on apamin-sensitive SK_Ca_ channels [55].

**Figure 7:**
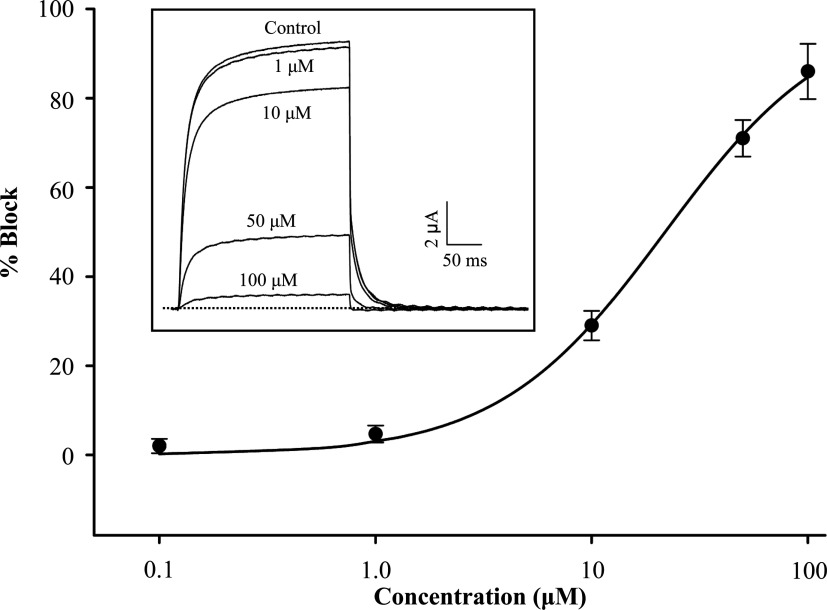
The dose–response curve of MCa on rK_v_1.1 Each point represents mean±S.E. (*n*=3). The representative whole-cell current traces in control and different concentrations of peptide conditions are shown in the inset. The dotted line indicates the zero-current level.

It has been reported that the lethality of MCa on mice after intracerebroventricular inoculation (LD_50_=20 μg/mouse) [56]; however, the molecular basis remains unsolved so far. Given key roles of K_v_1.1 in regulating action potential propagation and neurotransmitter release of mammalian brains [57], we evaluated in parallel the putative activity of MCa on this channel and found that it inhibited rK_v_1.1 currents expressed in *Xenopus* oocytes with an IC_50_ of 21.85±1.04 μM (Figure 7). This result on the one hand explains the lethality of MCa, on the other hand it suggests that K_v_ channels are a common target of two λ-KTx subgroups.

### Solution structure of λ-MK1_a_

Considering that there was only a limited amount of the full-length λ-MK1 available due to the C-terminal deletion during oxidative refolding (Figure 3A), we chose λ-MK1_a_ for solution NMR analysis in that the C-terminal amidation completely inhibited the non-enzymatic hydrolysis of the peptide and largely improved its refolding efficiency (Figure 3A). A similar CD spectrum between λ-MK1_a_ and λ-MK1 (Figure 4B) indicates that the modification does not significantly alter its structure.

**Figure 8:**
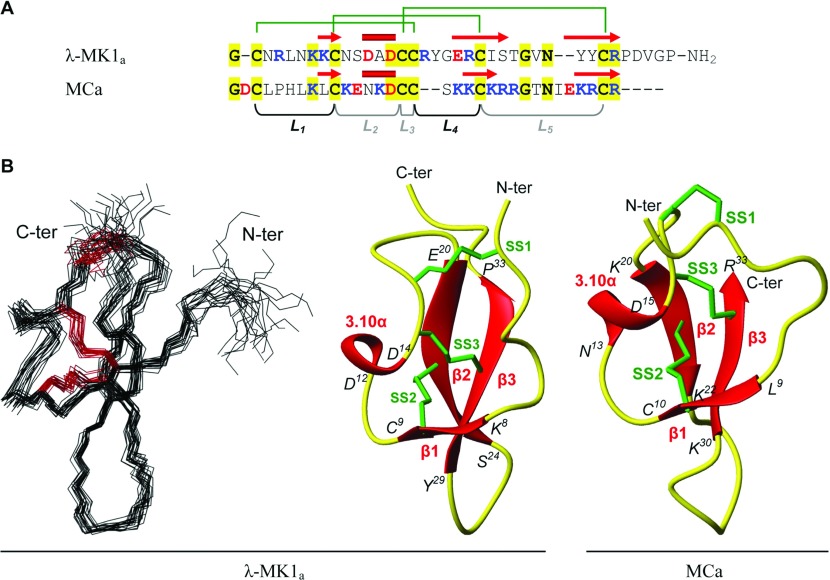
3D structure of λ-MK1_a_ (**A**) Structure-based sequence alignment between λ-MK1_a_ and MCa. Disulfide bridge connectivities and secondary structure elements (α-helix: cylinder; β-strand: arrow) were extracted from the coordinates of λ-MK1_a_ (this work) and MCa (pdb entry 1C6W); (**B**) Superimposition of backbone heavy atoms (N, C_α_ and C) for a family of 20 lowest energy NMR structures; a ribbon representation with disulfide connectivities shown in a stick model. The two termini are labelled with N-ter and C-ter and the strands (β) and helix (3.10α) are labelled in red. For comparison, the structure of MCa is also shown.

The 3D structure of λ-MK1_a_ was determined by NMR spectroscopy. The NMR spectra of λ-MK1_a_ showed good dispersion in the amide region, indicative of a highly structured peptide, and a full assignment was obtained. Amide temperature coefficients and deuterium exchange experiments were used to identify residues taking part in hydrogen-bonding interactions (Cys^9^, Asp^14^, Cys^15^, Arg^21^, Ile^23^, Arg^21^, Cys^31^, Tyr^32^). The 3D structure of λ-MK1_a_ was calculated with a total of 382 distance restraints, 69 dihedral angle restraints and 16 hydrogen bond restraints using a simulated annealing protocol in CNS. The disulfide connectivities (Cys^2^–Cys^16^, Cys^9^–Cys^22^, Cys^15^–Cys^31^) were also included as restraints in the structure calculations. The resulting family of structures (Figure 8) had good structural and energetic statistics, as shown in Table 1. The structures overlay well over the defined regions, with a RMSD for the backbone atoms of residues 2–34 of 0.73 Å. The overall Molprobity score (calculated as an average score of each of the structures) places the ensemble in the 82nd percentile relative to all other structures ranked by MolProbity. Analysis of the structures with PROMOTIF [58] identified a 3_10_-helix between residues 12–14 and a β-sheet comprising two anti-parallel strands between residues 20–24 and 29–33. Although not present in every one of the ensemble of structures, there is evidence for a small β-strand at residues 8–9. At other points in the peptide, a number of beta-turns were identified: a type II turn between towards the N-terminus between residues 4–7; a type VIII turn between the helix and the first major strand, residues 16–19; and a type IV turn forming a loop between the two major strands, residues 24–27. Superimposition of MCa [59] and λ-MK1_a_ revealed an RMSD of 1.91 Å across 32 C_α_ atoms, supporting their high structural similarity even when their sequences are substantially diverged.

**Table 1:** Experimental and structural statistics for 20 lowest energy structures of λ-MK1_a_

Parameter	Value
NMR distance and dihedral constraints	
Distance restraints	
Total NOE	382
Intra-residue	128
Inter-residue	254
Sequential (|i−j|)	122
Medium-range (|i−j|≤4)	37
Long-range (|i−j|≥5)	95
Hydrogen bonds*	16
Dihedral angle restraints	
Total	69
φ	28
Ψ	20
χ	21
Structure statistics†	
Violations	
Distance constraints (Å)	0.026±0.002
Dihedral angle constraints (°)	0.040±0.056
Deviations from idealized geometry	
Bond lengths (Å)	0.014±0.001
Bond angles (°)	1.34±0.04
Impropers (°)	1.58±0.15
Mean energies (kJ/mol)	
Overall	−1261±42
Bonds	24.8±2.4
Angles	59.8±4.7
Improper	24.3±3.8
van Der Waals	−144.0±5.9
NOE	0.28±0.05
cDih	0.04±0.08
Electrostatic	−1389±45
Average pairwise RMSD (Å)	
Backbone atoms (residues 2–34)	0.73±0.17
Heavy atoms (residues 2–34)	1.51±0.26
Stereochemical quality‡	
Ramachandran favoured (%)	91.9±1.9
Ramachandran outliers (%)	1.0±1.4
Unfavourable sidechain rotamers (%)	0.0±0.0
Clashscore, all atoms§	7.3±3.2
Overall MolProbity score	1.85±0.18
Percentile	82.2±7.4

*Two restraints were used per hydrogen bond.

†Statistics are given as mean±S.D.

‡According to MolProbity (http://molprobity.biochem.duke.edu).

§Defined as the number of steric overlaps>0.4 Å per thousand atoms.

### The interaction between λ-MK1_a_ and the channel pore: functional convergence among scorpion toxins with different folds?

It was previously known that most α-KTxs interact with the K_v_ channel pore by two crucial amino acids in a suitable distance: a conserved lysine side chain (i.e. Lys^27^ in kaliotoxin) protruding from the interaction surface of the toxin inserts into the channel pore to make intimate contact with the selectivity filter; and an aromatic amino acid (typically tyrosine or phenylalanine) at a 5–7 Ǻ distance from the lysine binds to the filter adjacent to the S6 helix by hydrophobic interaction [18]. Because of its presence in a broad range of structurally unrelated K^+^ channel toxins from various animals such as scorpions, cone snails, snakes and sea anemones, this dyad has been considered as a consequence of convergent evolution [18,60]. When analysing the structure of λ-MK1_a_, we found that the α-carbon of Arg^17^ and the centre of the aromatic ring (Tyr^18^) have an ideal distance of 5.73 Ǻ and these two highly exposed residues are conserved across the λ-KTx subgroup (Figure 2A), suggesting that they act as a functional dyad for toxin's binding. A putative dyad comprising an Arg and a Phe has been also proposed to explain the activity of APEKTx1 [51].

**Figure 9:**
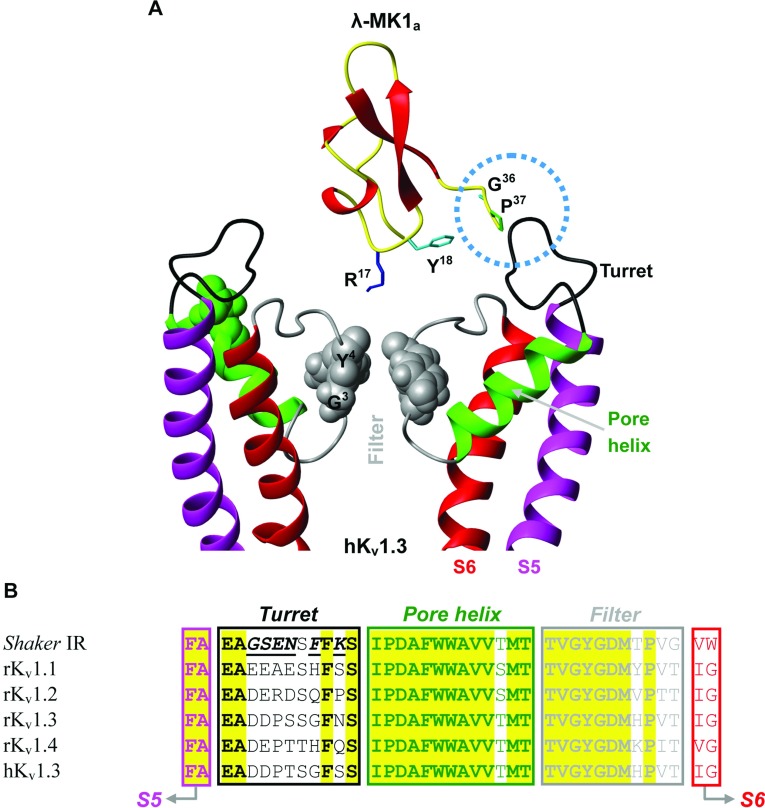
The putative functional surface of λ-MK1 (**A**) A possible interaction between λ-MK1 and the K_v_ channel, where Arg^17^, Tyr^18^ and Gly^36^/Pro^37^ are assumed to be implicated in direct binding to the channel pore; (**B**) Sequence alignment of the pore region of *Drosophila Shaker* and K_v_1.1–K_v_1.4 channels. *Shaker*-specific residues in the turret are italicized underlined once.

When the dyad was mapped on the interface between λ-MK1 _a_ and a K_v_ channel pore according to the mode of α-KTxs [61], we found that the functionally important C-terminus is close to the turret of the channel (Figure 9A) where there are six residues specific to the *Shaker* channel (Figure 9B) that could confer to the channel selectivity of this toxin. The conservations of the dyad and the C-terminal Gly–Pro in all the members of λ-KTx subgroup (Figure 2A) suggest that they constitute a common functional surface for K_v_ channel blockade. Our results provide further support for convergent evolution between scorpion K^+^ channel toxins with different folds. We also noted that MCa lacks the surface but still retains activity on Kv1.1. This suggests that members in the λ-KTx/calcine subgroup adopt a different mode to bind the channel, a phenomenon previously observed in the α-KTx group [62].

### Evolutionary divergence and ancient origin of animal ICK peptides

In this work, we have provided multidimensional evidence including precursor organization, exon–intron structure, 3D-fold and functional data, all support that the λ-KTx and λ-KTx/calcine subgroups originated from a common ancestor. The fact that all members in the λ-KTx subgroup are derived from buthidae, whereas all members in the λ-KTx/calcine subgroup are derived from non-buthids suggests that their divergence is associated with speciation of scorpions. Functional assays demonstrate that these two subgroups of toxins can target K^+^ channels, but only λ-KTx/calcine evolved the ability to activate Ca^2+^ release channels. Given the basal position of buthidae in the evolution of scorpions, it appears that the K_v_ channel-blocking activity was firstly developed in this ancient scaffold and then the Ca^2+^ release channel activating activity was evolved after the divergence of non-buthids from buthidae. In MCa, there are five contiguous basic amino acids associated with the second β-strand identified as its functional residues involved in activation of the skeletal Ca^2+^ release channels/ryanodine receptors [24], and all these basic residues are conserved in λ-KTx/calcine but obviously absent in λ-KTx (Figure 2A). This well explains the activity lack of λ-MK1 on the Ca^2+^ release channels/ryanodine receptors and provides new clues to understand how functional innovation occurred in a conserved structural scaffold.

**Figure 10:**
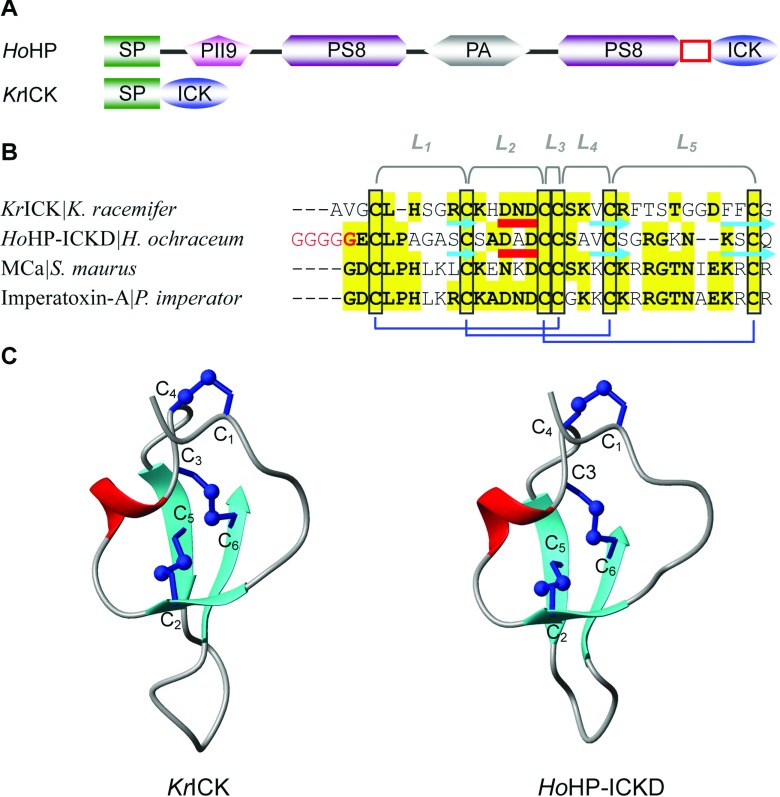
Bacterial ICK peptides (**A**) Schematic domain organization. Signal peptides were predicted by SignalP 3.0 HMM (http://www.cbs.dtu.dk/services/SignalP/). GenBank® accession numbers: *Ho*HP (*H. ochraceum* hypothetical protein): YP_003267557; *Kr*ICK (*K. racemifer* ICK): ZP_06968211. The glycine-rich hinge region is boxed in red; (**B**) Multiple sequence alignment. Conserved amino acids between bacterial and scorpion ICK peptides are highlighted in yellow; (**C**), Structural models. Ribbon representations showing overall folds of each peptide with disulfide connectivities in ball and stick format. The models were obtained by comparative modeling and the target-template alignment is shown in Supplementary Figure S1 (available at http://www.bioscirep.org/bsr/033/bsr033e047add.htm).

To study the evolutionary origin of ICK peptides, we carried out database search by using scorpion ICK peptides as queries. Consequently, we detected two bacterial peptides with a conserved cysteine arrangement pattern and highly similar amino acid sequence to the scorpion toxins (Figures 10A and 10B): one peptide (herein named *Ho*HP) is a hypothetic protein found in the bacterium *Haliangium ochraceum*, which is a multiple-domain of secretory protein including two PS8 (peptidase S8), one PII9 (peptidase inhibitor I9) and one PA (protease associated) domains and the C-terminal ICK domain (named *Ho*HP-ICKD); *Kr*ICK is a single-domain peptide composed of a signal peptide and a mature ICK, which is present in *Ktedonobacter racemifer*. *Ho*HP-ICKD and *Kr*ICK can adopt a typical ICK fold when the MCa structure was used as the template for homologous modelling (Figure 10C). These results indicate that eukaryotic ICK peptides are of ancient origin with a common ancestor prior to the eukaryote–prokaryote divergence (about 30 billion years ago) [63]. Further identification of these bacterial ICK peptides will provide new insights into the history of functional evolution of a conserved peptide scaffold.

### Conclusion

Although different venomous animals often use distinct scaffolds as templates to develop their own ‘pharmaceutical factory’, the ICK fold appears to be conserved across several evolutionarily distant venomous animal lineages. In cone snails and spider venoms, ICK toxins are predominant components and have rather diverse molecular targets, including Na^+^, K^+^, Ca^2+^, mechanosensitive and TRP channels [10,64–67]. In scorpions, ICK peptides only represent minor venom components and target a limited number of receptors (K^+^ channels and Ca^2+^ release channels). Given that scorpion has adopted multiple peptide folds to bind K^+^ channels, it is expected that many toxins currently found to have low potency on the K^+^ channels frequently used in the present assays will find their really native targets with high affinity. Alternatively, these weakly active components could play roles through allosteric effect with other highly toxic peptides in scorpion venoms [68]. Regardless of physiological roles of these toxins, the structural and functional characterization of the first scorpion venom ICK toxin with K^+^ channel-blocking activity sheds light on functionally divergent and convergent evolution of this conserved scaffold of ancient origin. In particular, the structure of λ-MK1_a_ represents the first experimental structure of the λ-KTx subgroup, providing valuable information to guide structure–function relationship research of this subgroup in the future.

## Online data

Supplementary data
