# Supplementary material for: Functional evolution of scorpion venom peptides with an inhibitor cystine knot fold
Source: Biosci Rep. 2013 Jun 27;33(3):e00047. doi: 10.1042/BSR20130052 (PMC3694633; doi:10.1042/BSR20130052)
Supplement: Supplementary data [file bsr033e047add.pdf]

Biosci. Rep. (2013) / **33** / art:e00047 / doi 10.1042/BSR20130052

OPEN ACCESS

Original Paper

# SUPPLEMENTARY DATA

# Functional evolution of scorpion venom peptides with an inhibitor cystine knot fold

Bin GAO*[1], Peta J. HARVEY†[1], David J. CRAIK†, Michel RONJAT‡, Michel DE WAARD‡ and Shunyi ZHU*[2]

*Group of Animal Innate Immunity, State Key Laboratory of Integrated Management of Pest Insects and Rodents, Institute of Zoology, Chinese Academy of Sciences, 1 Beichen West Road, Chaoyang District, Beijing 100101, People's Republic of China, †Institute for Molecular Bioscience, University of Queensland, Brisbane, Queensland 4072, Australia, and ‡Grenoble Institut des Neurosciences, Inserm U836, équipe 3, LabEx Ion Channel Science and Therapeutics, Bât. Edmond J. Safra, Université Joseph Fourier, Site Santé de la Tronche, BP 170, 38042 Grenoble Cedex 9, France

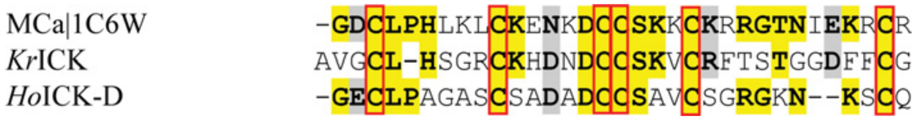


**Figure S1 Target-template sequence alignment for structural modelling**
Identical residues and conservative substitutions between targets and the template are shadowed in yellow and grey, respectively. Six conserved cysteines are boxed in red.

**Table S1 Primers used in the present study**
Note: R = A or G.

| Name | Sequence | Usage |
|---|---|---|
| MeuICK-F1 | 5′-ATGARTACTTTTRTCGTAGTC-3′ | 3′ RACE |
| MeuICK-F2 | 5′-TCTCACAGCTATTTTATGCCA-3′ | Genomic amplification |
| MeuICK-R | 5′-CCAGTGCTGATGCATCTCTCACCGT-3′ | 5′ RACE; genomic amplification |
| 3AP | 5′-CTGATCTAGAGGTACCGGATCC-3′ | 3′ RACE |
| dG | 5′-ATGAATTCGGGGGGGGGGGGGGG-3′ | 5′ RACE |

**Table S2 The expression of $K^+$ channel cDNAs in *Xenopus* oocytes**

| Clone | Vector | Restriction endonuclease | Promoter |
|---|---|---|---|
| $rK_v1.1$ | pGEM | *Sal* I | T7 |
| $rK_v1.2$ | pGEM | *Sal* I | T7 |
| $rK_v1.3$ | pGEM | *Sal* I | T7 |
| $rK_v1.4$ | pGEM | *Stu* I | T7 |
| *Shaker* IR | pBluescript | *Eco* RI | T7 |
| $rNa_v1.1$ | pLCT1 | *Not* I | T7 |
| $rNa_v1.2$ | pLCT1 | *Not* I | T7 |
| $rNa_v1.4$ | pBS SK+ | *Not* I | T7 |
| $rNa_v1.5$ | pSP64T | *Ase* I | SP6 |
| $mNa_v1.6$ | pLCT1 | *Not* I | T7 |

**Received 22 May 2013; accepted 23 May 2013**

**Published as Immediate Publication 30 May 2013, doi 10.1042/BSR20130052**

[1] These authors contributed equally to this work.
[2] To whom correspondence should be addressed (email Zhusy@ioz.ac.cn).

© 2013 The Author(s) This is an Open Access article distributed under the terms of the Creative Commons Attribution Licence (CC-BY) (http://creativecommons.org/licenses/by/3.0/) which permits unrestricted use, distribution and reproduction in any medium, provided the original work is properly cited.

Bioscience Reports www.bioscirep.org
